# Supplementary material for: Conducting active screening for human African trypanosomiasis with rapid diagnostic tests: The Guinean experience (2016–2021)
Source: PLoS Negl Trop Dis. 2024 Feb 20;18(2):e0011985. doi: 10.1371/journal.pntd.0011985 (PMC10906848; doi:10.1371/journal.pntd.0011985)
Supplement: Attachment — Submitted filename: PNTD-D-23-00775 Comments_25thSep2023.pdf [file pntd.0011985.s001.pdf]

PNTD-D-23-00775

Reviewer Comments

The authors present an interesting and helpful article that can inform practice and policy.

Methods

Include additional information on the study site regarding the prevalence and incidence of HAT so that your readers can understand the problem better.

How was the population sensitization done before sample collection?

Did you confirm that the sensitization was adequate?

How was the sampling/selection of volunteers done?

How did you avoid selection bias?

On putative cross-reactions in P. *falciparum*-infected participants, did you control for potential *hrp2/3* gene deletion?

Ethical Considerations

It is unclear if ethical clearance was sought before the medical surveillance campaigns. It would be good to include the name of the Institutional Research/Ethics Committee/Board. How did you ensure the confidentiality and privacy of the participants?

Was ethical approval sought before accessing the samples in the HAT-NCP biobank?

How did you determine the sample size for the HAT-NCP biobank samples?

In the various algorithms, how did you ensure the principle of beneficence, especially in participants whose screening tests turned negative and were asymptomatic or had atypical symptoms?

Results and Discussion

The findings are exciting and informative.

I'm unsure why you mixed the results and discussion, but you have lost some clarity. Perhaps you could have presented the results first, followed by a discussion bit, either for every key result or totally separate. For instance, the first paragraph in this section starts with statements

that sound like you are explaining something that should have been in the background or an explanation for a key finding.

Conclusion

The conclusion and recommendations are relevant. The study limitations are not clearly stated.
