# Supplementary material for: Conducting active screening for human African trypanosomiasis with rapid diagnostic tests: The Guinean experience (2016–2021)
Source: PLoS Negl Trop Dis. 2024 Feb 20;18(2):e0011985. doi: 10.1371/journal.pntd.0011985 (PMC10906848; doi:10.1371/journal.pntd.0011985)
Supplement: Attachment — Submitted filename: Response letter V1.pdf [file pntd.0011985.s004.pdf]

Point-by-point response letter to reviewers' comments

We are happy to read that all reviewers appreciated our manuscript. We thank them for their careful reading and their precise and useful suggestions. We have addressed their comments in the point-by-point response letter hereafter. The corresponding modifications are highlighted in yellow in the track-changes manuscript.

Methods

Reviewer #3:

Methods section has significantly improved, the descriptions are more clear.
Thank you.

The figure of the map is OK, but I think the quality should be improved, it is not possible to read the names of the provinces/foci, the font is blurry.
This is likely due to the file compression. The quality of the original figure that was uploaded to the submission website allows a comfortable reading of all legends.

It is not clear what when data collection for your analysis (study) ended, line 177 mentions january 2016 till May 2021 while line 222 talks about mid-2021 to date. please clarify if data collection ended may 2021 or december 2021?
The active screening campaigns took place in the three active HAT foci between January 2016 and October 2021. This was clarified l.177 and l.222.

As I am assuming that screening campaigns continue to this date.
Yes, of course. We are currently using the algorithm defined in the present study.

The last paragraph of your methods sections refers to tables, in which the results are already presented. To avoid confusion, I suggest not to refer to the tables yet, only when describing the results.
References to the result tables were removed from the methods section.

Line 232-237, you can finish your sentece by "RDTs were performed according manufactures instructions" you already explained briefly previously in lines 164-173, no need for repetion.
This was modified.

Lastly, in the results+discussion, you mention (434-437) analysis of costs (HR, transport, supplies...) however, it was not described in the methods how you determined these costs, where you collected and how you analysed.
The exact costs of all these campaigns were obtained from our financial office. The table comparing the budget details was removed from the first version of the manuscript as it was considered out of scope and not comprehensive enough. Here, we just aim at providing budget trends as an additional argument in favor of the final algorithm.

Results

Reviewer #3:

Combining Results and discussion is acceptable, in this case it did improve readability of the manuscript.

Line 341 is very confusing. Abbot 2.0 was only available from mid 2021 onwards, so its impossible to evaluate its performance over the 6 year period.
The 6-year period refers to the entire data set. We clearly mention in the text and tables that Abbot 2.0 was only tested for active screening between February and October 2021.

If I look at the tables and figures, Abbot was used in prospective observations for 6 months and also on all retrospective samples. But indeed the other RDTs were used during this 6 year period, so I'm assuming that you compared the performance of Abbot 2.0 (6 months) against other RDTs (6 years), am I correct? please clarify.
No, as detailed in the manuscript, SD Bioline HAT was used over 5 years and Sero-K-SeT for 3 years, but not continuously and with different algorithms. In total, Abbot 2.0 was tested over 9 months. The aim of this manuscript is to retrospectively compare, as fairly as possible, all these 3 RDTs between each other's and in combination, not only Abbot 2.0.

Paragraph starting line 410, indeed surprising that higher positivity rate was found in active screening. there was no hypothesis given for this? Line 415, your sentence starts with Similarly, however, the result is quite the opposite: positivity rate higher in active screening while PPV higher in passive screening? Did I interpret you text right? However, higher PPV in passive screening is to be expected, since people are presenting with specific clinical signs. Therefore your hypothesis nr 3 should come in first place. Also hypothesis nr 1, I could not understand the reasoning behind this explanation. Can you please clarify how malaria-positive patients are not further tested for HAT influence the PPV?
It is true that this paragraph was not well structured and possibly confusing. It has been reorganized and clarified according to these comments in the revised version.

As mentioned before, the results of the cost analysis (line 434) are not shown in a table or figure, or in supplementary info, but also was not described in methods how data collection was done and how analysis was done.
The exact costs of all these campaigns were obtained from our financial office. The table comparing the budget details was removed from the first version of the manuscript as it was considered out of scope and not comprehensive enough. Here, we just aim at providing budget trends as an additional argument in favor of the final algorithm.

Conclusions

Reviewer #3:

The final conclusion is acceptable, it would also have been noteworthy to mention what the best screening algorithm would be in passive screening.
Yes, this will likely be the object of another study.

Editorial and Data Presentation

Reviewer #1:

Yes it is adequate, although I still prefer to see results and discussion seperate, but leave this to the editor.
Yes, but again, after several tries, this way is by far the clearest for presenting this complex data set.

Summary and General Comments

Reviewer #3:

The manuscript has significantly improved, however, there are minor issues that still need to be addressed before acceptance.
All reviewer's comments were considered for improving further the manuscript.

Reviewer #4:

The authors have provided a thorough response to the comments by myself and the other reviews, making substantial changes to the manuscript to include additional details (especially with regards to ethical approval) and clarify the presentation. The table and figures are much improved and far more legible than was provided in the original submission.
At this point I am happy to recommend the manuscript for publication as while the work is not groundbreaking it is nonetheless valuable as the data will guide public health policy going forward and directly aid in the elimination of trypanosomiasis in the future.
Thank you.
