# Supplementary material for: Conducting active screening for human African trypanosomiasis with rapid diagnostic tests: The Guinean experience (2016–2021)
Source: PLoS Negl Trop Dis. 2024 Feb 20;18(2):e0011985. doi: 10.1371/journal.pntd.0011985 (PMC10906848; doi:10.1371/journal.pntd.0011985)
Supplement: Attachment — Submitted filename: Response letter V3.docx [file pntd.0011985.s003.docx]

**Point-by-point response letter to reviewers’ comments**

We are happy to read that all reviewers appreciated our manuscript. We thank them for their careful reading and their precise and useful suggestions. We have addressed their comments in the point-by-point response letter hereafter. The corresponding modifications are highlighted in yellow in the track-changes manuscript.

# Methods

**Reviewer 2:**

Include additional information on the study site regarding the prevalence and incidence of HAT so that your readers can understand the problem better.

How was the population sensitization done before sample collection?

Additional information was included in the revision (section ‘HAT serological screening strategies during door-to-door campaigns’).

Did you confirm that the sensitization was effective?

Yes, sensitization / awareness raising was effective because all volunteers provided their individual signed informed consent, the content of which was explained by the researchers or health workers involved in the study in the presence of a witness before sample collection.

How was the sampling/selection of volunteers done?

How did you avoid selection bias?

Additional information was included in the revision (section ‘HAT serological screening strategies during door-to-door campaigns’).

On putative cross-reactions in *P. falciparum* infected participants, did you control for potential hrp2/3 gene deletion?

No, this is indeed a good question but that probably goes out of the scope of the present paper. Hence, without any evidence, we have carefully used the term ‘malaria-related biological factors’.

Ethical Considerations. It is unclear if ethical clearance was sought before the medical surveillance campaigns.

Yes, of course. As explained in the dedicated section, ethical clearance was sought before this study to obtain a long-term authorization from the Comité National d’Ethique en Recherche Scientifique de Guinée (CNERS) attributed to the HAT-NCP of Guinea.

It would be good to include the name of the Institutional Research/Ethics Committee/Board.

Yes, this is the Comité National d’Ethique en Recherche Scientifique de Guinée (CNERS) that is now clearly mentioned in the ‘Ethical Considerations’ section.

How did you ensure the confidentiality and privacy of the participants?

After obtention of an individual informed consent, each participant was attributed a unique code / identifier (LLNNNN/N) in order to anonymize all the next steps and ensure the confidentiality and privacy. A researcher or MD was in charge of this task during each campaign. This is now clearly mentioned in the ‘Ethical Considerations’ section.

Was ethical approval sought before accessing the samples in the HAT-NCP biobank?

Yes, of course. As explained in the dedicated section of the material and methods: ‘For the retrospective analyses, all sera originated from the HAT-NCP Biobank built from studies where participants or their legal representatives signed an individual informed consent before enrolment (Studies Diag-Cut-THA 032/CNERS/17 and amendment 038/CNERS/19).’

How did you determine the sample size for the HAT-NCP biobank samples?

Additional information was included in the revision (section ‘Retrospective analyses on samples from the HAT-NCP Biobank’).

In the various algorithms, how did you ensure the principle of beneficence, especially in participants whose screening tests turned negative and were asymptomatic or had atypical symptoms?

This study is mostly based on retrospective analyses of the data collected during the routine activities of the HAT-NCP in Guinea. Active screening campaigns aim at detecting all cases for systematic treatment in target populations. Individuals with negative screening tests and no symptom (asymptomatic) are considered negative for HAT. Individuals with negative screening tests and atypical symptoms can be sometimes further examined according to the decision of the MD in charge of the campaign and based on the clinical picture and the epidemiological risk evaluation. Individuals with negative screening tests and typical symptoms, especially swollen cervical lymph nodes, are systematically referred to the main lab for parasitological investigations.

**Reviewer #3:**

Yes, the objectives of the study are clear, however, the manuscript lacks a bit of structure, some parts are very confusing for the reader and some descriptions need more specific details.

After several tries, we decided to combine the results and discussion sections for a better efficiency and clarity.

**Reviewer #4:**

The materials and methods are clearly described and appropriate to the study, with a focus on

assessing the sensitivity and specificity of the respective RDTs in active HAT foci following a change in the availability of the SD bioline HAT RDT. The study procedures are clearly described, and the use of a retrospective analysis provides the opportunity to directly compare the tests using samples whose infection status has already been determined. Ethical considerations are clearly stated and were approved by the appropriate national bodies.

Thank you

# Results

**Reviewer #2:**

The findings are really interesting and informative. I’m not sure why you mixed the results and discussion, but you seem to have lost some clarity. Perhaps you could have presented the results first, followed by a discussion bit, either for every key result or totally separate. For instance, the first paragraph in this section starts with statements that sound like you are explaining something that should have been in the background or an explanation for a key finding.

This is what we have tried at first, but we decided to combine the results and discussion sections for clarity reasons, as the complex chronology of the multiple algorithms was not easy to grasp and connect when both parts were separated.

**Reviewer #3:**

The results should be re-structured a bit, double check figures and tables if they are correctly numbered and refer to the correct tables and figures.

We have carefully rechecked the table and figure numbers.

**Reviewer #4:**

The authors present a clear summary of the results, broken down into appropriate sections. The retrospective analysis comparing the three RDTs and cross reactivity survey are of particular interest as they highlight the key results and challenges faced during screening in a malaria endemic region. The identification of possible cross reactivity with malaria antigens warrants further investigation, especially as HAT cases continue to decline in prevalence. The figures are basic but clear and appropriate to the results.

Thank you

The tables, which present the core results, would benefit from some revision to improve legibility as the included in line versions are far too small. The authors may wish to consider moving some details to the supplementary section and focus on only a small number of the currently included columns in the main text.

After editing by the PLoS staff prior publication, the tables will be presented in full pages in the optimal orientation to allow an appropriate reading. We think that all the data presented in these tables are important to understand and compare the different screening conditions and results.

With the exception of Figure 1 there are limited figure/table legends and more details are required here.

Legends were improved in the revised version.

# Conclusions

**Reviewer #2:**

The conclusion and recommendations are relevant. The study limitations are not clearly stated.

This study is mostly based on retrospective analyses of the data collected during the routine activities of the HAT-NCP in Guinea. Its limitations are detailed in all tables: different screening algorithms had to be used in different combinations according to the availability of the different RDTs.

**Reviewer #4:**

The conclusions drawn throughout the combined results/discussion are appropriate and clearly presented. The authors discuss potential reasons for the differences observed between the RDTs and the differences observed between this sample and the higher sensitivities observed in the DiTECT-HAT clinical trial. The public health relevance is clearly discussed throughout, from both the perspective of sensitivity/specificity and resource costs that can be saved by using the combination algorithm described in the manuscript. They highlight an in progress study that will extend these results to both currently used RDTs and new prototypes

designed for D2D screening.

Thank you

# Editorial and Data Presentation

**Reviewer #4:**

The study is clearly written throughout and overall well presented. As already noted some of tables are too dense and will require reformatting to use either a full page design or a simplification to enable the most important details to be clearly visible. This revision is should not be hard to make.

We agree that the current presentation of the tables is not adapted. For an easier review, you may download each table independently by using the links provided by PLoS. After editing by the PLoS staff prior publication, the tables will be presented in full pages in the optimal orientation to allow an appropriate reading.

# Summary and General Comments

**Reviewer #1:**

This manuscript is addressing an important issue in neglected tropical diseases, i.e. the correct

diagnosis of HAT. The manuscript is of interest for the public of this journal. In principle the research is sufficiently designed and presented.

Thank you

There are however issues that require the attention of the authors and that need to be resolved.

The use of English requires some attention; there are some minor errors found within the manuscript. Possibly this can be done through an editorial check.

Yes, the revised manuscript has been rechecked for English and an additional reading will certainly be done by the PLoS editors.

An important issue is the ethical consideration. There seems to be no appropriate review and approval by competent ethical review bodies in the country concerned. Furthermore, although an informed consent form has been signed by participants, probably at the time of signature they were not informed about the current study. This is an issue and must be resolved.

We agree that this is an important aspect and some more details on ethical clearance are now provided in the revised version. See the ‘Ethical considerations’ and ‘HAT serological screening strategies during door-to-door campaigns’ sections

The methodology section is rather detailed and could be shortened. For example, lines 195-202 are much repeating lines 142-153 (although the first addressed the case of no consensus).

We think that providing a detailed method section is important here. Hence, as requested by the reviewer, we added some more details on ethical aspects.

The methods described lines 142-153 and 195-202 are referring to distinct conditions: the field protocol and the lab assays, respectively. The biobank sample volume being limited, we had to adapt the protocol by reducing the tested volumes to be able to run all RDTs in parallel. We thought it was important to detail these differences.

If possible batch numbers and expiration dates of tests used should be provided.

Unfortunately, this will not be possible.

Were any positive/negative control samples used during the campaigns to ensure proper use of the RDTs?

No, providing this kind of QC samples to each team in the field for each activity is logistically difficult to organize. However, prior to each door-to-door campaign, a compulsory half-day training and rehearsal session was systematically organized for all the health workers involved.

The retrospective analyses in samples from the Biobank raise some questions. It seems that plasma (or sera?) have been used from (non) cases; this needs to be clarified.

We tested plasma samples collected from clinical and / or serological RDT suspects, that were parasitologicaly confirmed (cases) or not (controls, CATTwb-) prior cryobanking.

Use of blood seems to be the preferred diagnostic sample (see lines 143 – 147). Could the use of plasma/sera affect the test performance?

The RDT manufacturers claim that their tests can be used with human whole blood, serum or plasma. Here, we tested all RDTs in parallel with the same plasma samples in the same conditions to allow their comparison.

Samples of CATT negative controls were used. Did these comprise other endemic diseases, like malaria, as this seems to be a concern.

Tests for other endemic diseases, including malaria, are not systematically performed in routine by the HAT-NCP teams because of the vertical organisation of the national control programs in Guinea (this is however currently evolving in a good direction). Only the subjects enrolled in the sub-study described in Table 5 were all tested for both HAT and malaria in parallel.

In the section statistical analysis it is mentioned that sensitivity/specificity were calculated on confirmed HAT cases. How were these cases confirmed? By parasitology? Or was it only serology (in that case, I have some doubts about the accuracy).

According to the HAT-NCP guidelines, HAT cases are necessarily confirmed by parasitology.

The tables must be modified to ensure unity of style. Currently many different lay-outs (and often hard to read) are being used. Headings to these tables should be more descriptive.

We agree that the current presentation of the tables is not adapted. The tables will be homogenized and presented in full pages in the optimal orientation by the PLoS staff prior publication.

The so-called cost-effectiveness should be removed from the manuscript. CE calculations require a much more in-depth study design and the current work is just a very rough description.

Yes, we agree. Table 6 was removed, and a short description of the key ideas was added in the revised discussion.

For clarity reasons, I would propose to separate results and discussion into two separate sections.

This is what we have tried at first, but we decided to combine the results and discussion sections for a clarity reasons, as the complex chronology of the multiple algorithms was not easy to grasp and connect when both parts were separated.

**Reviewer #2:**

The study topic is helpful. However, the authors need to improve the methods section to assure readers that ethical principles were satisfactorily considered.

We agree that this is an important aspect and some more details on ethical clearance are now provided in the revised version. See the ‘Ethical considerations’ and ‘HAT serological screening strategies during door-to-door campaigns’ sections.

**Reviewer #3:**

dear Authors,

The manuscript describes an interesting topic that is gaining more importance for the diagnosis of HAT.

There is an overall lack of structure in the way the results are described and presented in the manuscript, making it difficult to understand for the reader.

Detailed comments can be found in the attachement.

Thank you very much for these detailed comments that were carefully considered for the revised version. We decided to combine the results and discussion sections for clarity reasons, as the complex chronology of the multiple algorithms was not easy to grasp and connect when both parts were separated.

**Reviewer #4:**

The study by Camara et al aims to compare the sensitivity and specificity of the RDTs that have been used within Guinea to identify HAT infections as the country moves towards elimination trypanosomiasis as a public health concern. The study combines current datasets with a retrospective analysis in an appropriate manner that allows for a direct comparison of the three RDTs being studied. The study is relatively simple in design and not particularly novel but addresses an important question that has clear public health and policy implications as countries push towards the elimination of HAT, hence my recommendation that it is accepted

with only minor revisions.

Thank you
